# Lost futures, doomed timelines and unwanted inheritances: how we are handling painful time in social work

**DOI:** 10.1332/204986022X16703011487757

**Published:** 2023-01-06

**Authors:** Stephanie Davies

**Affiliations:** Birkbeck, University of London

## Abstract

This article makes the submission that social work is stuck and needs now to find ways to endure its commitments to caring from inside the suspended time that is so characteristic of late capitalism and not from some imaginary place outside of it. When telling this time in the form of history, there is a tendency to want to pass over what is most difficult about it – the inescapable fact of having to live through it – just at the moment when this is the reality most in need of being carefully thought about. Remembering that in talking about social work, we are talking about a labour of care defined, in part, by a sensitive, practical engagement with time that is difficult to live, I look to recent feminist theoretical work on care that can help us to think about how we might handle being stuck in painful time.

## Introduction

Contemporary histories of social work will often tell the story of the past as a way of retracing steps and trying to work out what has gone wrong in the present (Rogowski, 2010; Chapman & Withers, 2019; Brady, Sawyer and Perkins, 2019). They constitute a ‘now time’ in which social work is defined mainly by its quality of being stuck or suspended, or in an impassable state of being ‘at a crossroads’ (Lavalette, 2011). With this essay, I have no solution to offer for the problem of being stuck in a late, protracted historical present, whether this is due to having to wait for the passing of the latest stages of capitalism (Jameson, 1984; Fisher, 2009), or the last dregs of a late liberalism (Povinelli, 2021). What I would like to do, is to try to pose the question of how as social workers, we are handling this stuck time which some have described as ‘lacking in hope’ (Rogowski, 2010:164), others as ‘difficult to live’ (Chapman & Withers, 2019), and which I have described as ‘painful’ for reasons that will become apparent.

The first section of this paper provides a background to my question by introducing three social work histories all published within the last twelve years. They culminate in the loss of a promised future (Rogowski, 2010), in the realisation of having helped to progress a doomed timeline (Chapman & Withers, 2019), and in the desire to cut ties with an unwanted inheritance (Brady et al., 2019). I look at each one of them in turn to see how they constitute their own distinct version of a social work present, and find that their primary problem is the shared predicament of being stuck in some way and barred from moving on. In the second half of the paper, I try to think about this from inside the suspended time that is so characteristic of social care labour in the present. In the everyday life of a social worker, discontinuities, breaks and breakdowns easily prevail over the fantasy of a central progressive timeline. Relocating social work from its sticking point at the ‘end of history’ to the phenomenal present of everyday life, I look to feminist theoretical work on care, particularly that of Maria Puig de la Bellacasa and Lisa Baraitser, which can help us to think about how we might handle time that we may not want to live because it ‘hurts’ (Berlant, 2011), but which we must, if we care.

## A lost future

…to cut a long story short, the dominance of neoliberalism remains and the economic and political system this entails has emerged from the crisis largely unscathed. This is the reason why we have surely seen both the changing face of social work and arguably its fall.

(Rogowski, 2010:183)

Rogowski is well known for his persistence in defending the project of social work in England in late times. For instance, he has argued for the use of critical practices when working with older people, especially because we are still living in ‘neoliberal times’ (Hastings & Rogowski, 2015: 21). He has suggested approaches to working with young people that ‘resist the neoliberal present’ (Rogowski 2013:7). Most recently, he has searched out opportunities in social work with children and families to keep supplying the ‘much-needed antidote to the unequal neoliberal world we currently inhabit’ (Rogowski, 2021:353). With this in mind, his history of social work which was published some time ago now in 2010, represents something of a departure from the usual tone of dogged optimism that has characterised so much of his work. At the time of writing it, it is clear that Rogowski still believed in social work’s potential for making a better world, ‘one where at the very least there is less inequality and less oppression’ (Rogowski, 2010:8). However, there is also a tragic quality to his reflections on social work’s past and a sense of something of great value having being lost, perhaps irretrievably.

The story he tells is a familiar one. It comes dangerously close in fact to offering quite a good example of what has elsewhere been branded as the ‘standard account’ of social work in the West. According to some, this is an account that needs ‘troubling’ (Chapman & Withers, 2019:25). Rogowski’s history of the profession can be summarised as follows: social work in England originated around the middle of the 19th century. To begin with, there were the tentative, hopeful attempts at a new beginning ‘when the state, often reluctantly, became more involved in welfare provision’ (Rogowski, 2010:10). Then, from the middle of the 20th century onwards, the advancement of a welfare state helped to consolidate social work as a profession. The welfare state goes from strength to strength, seeding many good projects of care and social development until the 1970s. This is the turning point when a change of political direction brings an end to the democratic consensus that had helped social work to flourish. This is also the decade when Rogowski was initiated into the profession and the period that he thinks of in retrospect, as the time when social work had ‘reached its zenith’ (Rogowski, 2010:10). Then begins the heavy, downward turn from the 1980s onwards when social work started to drift further and further away from its original hopeful destiny. For those of his generation who feature in the book as contemporaries and interlocutors (Beresford & Croft, 2004; Lavalette, 2011; Reisch, 2002), this marks the beginning of a long struggle to preserve all that had once seemed self-evidently ‘good’ about social work. For Rogowski who continues to practise during this time, this has meant more than four decades of defending the integrity of an earlier model of social work alongside having no choice but to implement the changes that will make this into a thing of the past. At the end of it all, there is very little that actually remains of the world that Rogowski had tried so long to keep going. He sums this up as ‘rationing and risk assessment, which has to be carried out as speedily as possible under the direction of managers’ (2010:23).

*Social Work: The Rise and Fall of a Profession?* is very much the personal history of having lived through years of ‘swimming against the tide’ and ‘bemoaning the changes that have taken place in social work’ (Rogowski, 2010:4). My lasting impression is of Rogowski’s grief at the thought of losing something that is felt to be so much needed in this time and of his understandable wish to hold onto it for a little while longer. Lost as it may be, he cannot just let go of the idea that a truly good version of social work remains possible at some future point. In spite of everything, he holds onto the idea, that ‘a niche can be found’ and that social work will have the chance of surviving the neoliberal present (Rogowski, 2010:187). Possibly as a consequence of this, two versions of social work in the present emerge, neither of them quite fully here, but not gone yet either. There is the social work which is at best a ‘limited version of what the possibilities once were’ (Rogowski, 2010:21), and there is the social work whose demise we are now witnessing because it has no place in ‘the individualistic world in which we currently live’ (2010:25). Real social work exists, but only as something to be recovered from the past.

## A doomed timeline

How can we hold our responsibility to challenge capitalism, colonialism, the institutionalization of human relationships, the depoliticization of social problems, and the forces of interlocking oppression alongside the recognition that we’re complicit in these systems and will likely perpetuate them? Living this contradiction is difficult.

(Chapman & Withers, 2019:355)

In *A Violent History of Benevolence,* Chapman and Withers want to raise the moral consciousness of the profession as a whole by making social workers more aware of how they are deeply implicated in systems of oppression dating back hundreds of years. Locating social work as a discourse of colonialism, they place its origins firmly within the time of white European Imperialism. This book is intended both as a corrective to the false view that ‘things are getting better through the gradual implementation of social reforms’ (Chapman & Withers, 2019: 323), and also as a tool to shock social workers into questioning what they are doing. By situating social work in an alternative pessimistic history, they re-envision its practices as the extension of forms of violence that began when white colonizers imagined themselves to be creating a new world governed by reason at the start of the crusades in the eleventh century. The far-reaching claim here is that that there is something intrinsically violent and doomed about a project originating out of the ‘oppressive legacies’ and ‘historical violences’ of colonizing social work practices (Chapman & Withers, 2019:4). From such a perspective, social work is made to appear particularly insidious because it carries out an imperial project of progression concealed in practices of care. Its violence cannot be confined to the past as some people might prefer to believe, because wherever the two activities of care and progress are enjoined (as they almost always are in professional social work), its specific brand of violent benevolence is reproduced. This is how the belief in progressive social work comes to be described as toxic, harmful and capable of ‘real Damage’ (Chapman & Withers, 2019: 333). Following Michel Foucault’s (1972) genealogical method of tracking the power that ‘passes through individuals’ (Foucault, 2003:29) because of how they are ‘totally imprinted by history’ (Foucault, 1980:83), Chapman and Withers argue that there is an inevitability to the harm that social workers have always done, always do and will always continue to do. As the opening line of the book makes clear, this is the logic of ‘history produces the present’ (2019:3). The potential for this particular form of ethical irresponsibility is believed to be internal to the social work ethic itself, summed up as ‘the healing power of domination and imagined moral superiority’ (2019:366).

One way of staying orientated when moving between different, conflicting accounts of social work’s past is to look at how each author tries to engage ideas about progress and progressiveness. For Rogowski, it has become more difficult, impossible even, to go on believing that social work in its current state could still be helping to create a better future for all. His memory of the last good time in the profession’s history was when there was still some optimism about the role it would play in helping to build the British welfare state, a project that he sees as being ‘congruent with the Enlightenment project’ (Rogowski, 2010:22). For Chapman & Withers, the belief in a progressive and benevolent welfare state forms part of the same system of belief that has prevented social workers from becoming fully conscious of the harm they do. The initiation of social workers into ‘knowing about social work’s violences without ever imagining that they may also be complicit in them’ allows them to go on believing that they are morally superior (Chapman & Withers, 2019: i). This is a horrific assessment of social work reaching back centuries: social workers believing they are helping people to escape or move on from histories of oppression, when in reality all they are doing is prolonging and perpetuating colonial violence.

## An unwanted inheritance

Despite the many contributions of social work and social workers, it is tenuous to claim that the profession of social work has emphasised or does emphasise radical change.

(Brady, Sawyer & Perkins, 2019:382)

As the name of their paper suggests, in Debunking the Myth of the Radical Profession, Brady, Sawyer & Perkins set out to debunk as myth, the notion that there has ever been any legacy of radical social work in the US, the UK, Canada or Australia. Though broadly supportive of social work as a useful institution in the US where they are based, they reject the claim that professional social work has had any significant part to play in the history of radical social movements in the West. As they put it, ‘little evidence confirms that the social work profession ever focused substantially on transforming societal systems, challenged the status quo culture or dismantled dominant narratives (Brady et al, 2019:13). Their proposal is that by embracing the reality of social work as a profession that works closely with social systems to maintain the status quo, ‘we can focus on re-visioning ourselves as a more critical, progressive and advocacy-oriented profession’ (Brady et al., 2019:14). The claim is that social work has never been radical at *any* point in its history; that it has ‘seldom stood opposed to large-scale injustices and taken unified radical concrete actions or focused on transforming oppressive systems’ (Brady et al., 2019:13). This is not about social work having failed in its ambition to be a radical project of social change, a charge which would be nothing new or surprising in itself (Rogowski 2010; Reisch and Andrews, 2002). It is a proposal for how we might read the standard history of social work differently by suspending belief in the idea that social work has ever had a commitment to anything transcending its daily grind in the neoliberal present. Such a disavowal of any and all radical legacies goes beyond a rejection of what may be factually incorrect. The only reality that the authors will concede to imagining for social work at any time in its history is a neoliberal one. This is where social change is ground out so extremely slowly through the workings of large, conservative bureaucracies over successive generations that when something finally does happen, as when for instance, a progressive policy is introduced, this only amounts to what would be considered reasonable and in keeping with the time.

When trying to understand why, as social work educators themselves, the authors of this work might want to reject the idea ‘that social work is or ever has been a profession deeply committed to radical social change’ (Brady et al., 2019:2), it feels very relevant to look at the generational differences that exist within the profession today. For those (myself included), whose entry into social work dates no further back into the past than thirty years, the radical histories that we inherit are stories that have been passed down without actually having been lived. Superficially they may enliven social work as a site for optimism, but there is no reference for them in living memory. An attachment to the radical elements of social work history poses a problem for those who have only ever known or experienced social work in the neoliberal present. Not only are its projections for social transformation made to appear unrealistic. They can be made to seem as though out of synch with reality.^1^ As we have already seen, to be on the receiving end of such an inheritance can lead to despair, guilt and paralysis (Rogowski, 2010; Chapman & Withers, 2019). Social workers can find themselves in an ‘invidious position’ where they are required to conform to a set of political conditions ‘in a manner anathema to what have been social work principles’ (Wallace and Pease, 2011:135). Also, for those who do not possess any living memory of it, the past of social work may not offer the same resources of hope mixed in with despair as it might for those who do. To disinvest from the past as a major site of optimism could be considered desirable under the circumstances and perhaps even necessary if the profession is to move on.

## The stuck social work present

In each of these three narrated histories: a lost future, a doomed timeline and an unwanted inheritance, the social work present is constituted as a time that has become too dangerous to live in its current form. The widely shared belief in its hazardousness may come in part as a consequence of discontinuity and in the difficulty for social workers of finding a way of going on from here. For Rogowski, social work in the present is frighteningly duplicitous to the point that we don’t know exactly what we are dealing with anymore. He uses Green’s (2009) label of ‘deformation’ to describe its current state, reminding us again and again that ‘what remains of social work is a limited version of what the possibilities once were’ (Rogowski, 2010:21). It is a constant source of perplexity to keep finding that the social work he once knew appears to have been outmoded by the times he is living in. He wants to know where is he now? What is *this*? Is this ‘the changing face or the fall of social work?’ (Rogowski, 2010: 161); ‘have we in effect witnessed its fall as a profession?’ (2010: 162); ‘is there a future for social work?’ (2010:181). For Chapman and Withers, the present takes the form of ’a contradiction’ that is ‘difficult to live’ (Chapman and Withers, 2019:356). One of their aims in foregrounding the violence of social work is to compel social workers to question the morality of what they are doing, to the point that they feel paralysed by doubt and ‘no longer know what to do’ (Foucault, 1991). Paradoxically, this could make it more dangerous for them to acknowledge their complicity in oppressive systems. They may find that they ‘get so caught up in the fear they’ll cause harm that they never do anything concrete’ (Chapman and Withers, 2019: 356).

The anthropologist Ghassan Hage uses the term ‘stuckedness’ to describe the condition of people whose situation in life has led to them becoming convinced that any further progress in the form of forward movement is now barred to them (Hage, 2009). If the belief that one is going somewhere (or could go somewhere if they wanted to) is necessary for life to feel viable, then the realisation that they are not going anywhere could lead to life being experienced as something unviable, i.e., as lacking in what life needs to be lived well. When allied to a crisis situation, being stuck implies that an unwanted duration of time is having to be waited out before the arrival of something better. ‘Waiting out’, Hage explains, ‘is a specific form of waiting where one is not waiting for something but rather waiting for something undesirable that has come, like a spell of cold weather or a disliked guest, to end or to go’ (Hage, 2009:102). Unlike waiting that can be passive or active, waiting out is always passive. It involves both ‘a subjection to the elements or to certain social conditions and at the same time a braving of these conditions’ (2009:102). For social workers, as for others in the welfare sector, finding yourself in the situation of waiting out bad working conditions for indefinite lengths of time is bound to have consequences for how caring engagements can be formed and sustained. If the original project of collectively funded social care is made to appear unviable, then social workers could be obliged to find other ways of making some kind of success out of their work. Even assuming that their stuckedness could be looked upon as an achievement in a situation in which, as Chapman & Withers argue, it would be more terrible just to keep going, practising social workers presumably have to find some way of getting on with things. They cannot suspend their caring activities in the present any more than they can suspend having to be accountable for them. So, what does it mean to ‘go on’ like this, without being able to ‘move on’ from being stuck? How is a person supposed to ‘do’ social work when they no longer know what to ‘do’? The predicament of having to keep going after having lost a secure footing in relation to both the past and future is not exclusive to social work. For the cultural theorist Lauren Berlant (whose death was announced during the writing of this paper), this is just part of what is involved in living the turbulence of the neoliberal present. Much of Berlant’s work is an attempt at unpicking the many ways in which we are always ‘catching up’ with the historic and historical crisis that suffuses our time. In *Cruel Optimism,* they constitute it as ‘a space of transition, not only between modes of production and modes of life, but between different animating, sustaining fantasies’ (Berlant, 2011:261). For those of us whose whole lives continue to be intertwined with a social democratic vision of welfare in both real and imagined ways, a perception of having lost it as a living thing, could amount to feeling ourselves to be living after the end of some kind of history.

In Freud’s formulation, melancholia can occur as a reaction when ‘the object has perhaps not actually died, but has been lost as an object of love’ or when something or someone may have died, but ‘one cannot see clearly what it is that has been lost’ (Freud, 1917/1957:245). Melancholia is compared by Freud with the experience of mourning as they share some of the same traits, including ‘a profoundly painful dejection, cessation of interest in the outside world, loss of the ability to love’, sometimes also accompanied by ‘a lowering of the self-regarding feelings to a degree that finds utterance in self reproaches’ (1917/1957:244). Interestingly, the sociologist Shona Hunter uses melancholia as a device for understanding what it might mean for British public sector workers to be living through the loss of certain ‘objects’^2^ that they have come to depend on for their own sense of identity and direction (Hunter, 2015). Posing the political past as a lost fantasy object is tricky and questionable (Fisher, 2014), but if handled carefully, melancholia understood not so much as a psychological state but as a heuristic device may be helpful for thinking about why, for the time being at least, it may not be realistic to expect that such a loss could simply be processed or ‘gotten over’ (Hunter, 2015:4). The lost or failed objects in this case are those that belong to the idea of the Western style social democratic welfare state with its particular formations of solidarity, progress and hope for the future. As complicated as the history of the state may be with its harms and hopes always interlocking, it is still the main way that social workers have of reproducing themselves and their contributions to caring for the social. The difficulty of knowing what to do with failed objects, including ‘Progress’, is that if who and what we are today is basically a continuation of the liberal democratic vision, then ‘to abandon it would be to abandon ourselves’ (Hunter, 2015:17). Uncomfortably, this situates whole generations of public sector workers somewhere between the loss of the thing that has sustained them, and self-abandonment at the end of their working lives. At a time when they might have expected they would be coming to the completion of something worthwhile, they can find themselves stuck in a state of perpetual ambivalence towards the most basic assumptions about what they have worked for all those years.

## The persistence of care in everyday life

So far, I have said very little about that part of social work that we would normally refer to as care, which is not so much an event in time as it is a labour that takes time. It might be what most social workers are labouring towards, and yet the realities of care are often neglected in histories of social work. In this next section, drawing from the work of contemporary feminist theorists of care, Maria Puig de la Bellacasa and Lisa Baraitser in particular, I try to shift the temporal axes of the debate so that instead of trying to define itself in relation to an ‘open libidinal future’ (Baraitser, 2017:61), social work is defined instead by what it already produces and perpetuates through its daily labours. To make the shift from care as a moral imperative to care as a relation, requires a changeover of temporality from the national narrative to the ‘persistence of the everyday’ (Harootunian, 2004). It also requires re-attunement to the social care world as one in which the work of care for the social is open-ended and non-idealised, arising out of an attachment to things cared for and about, rather than in response to the moral imperative to care for them. This is the re-attunement that tries to make care ‘unthinkable as something abstracted from its situatedness’ (Puig de la Bellacasa, 2017:6). Puig de la Bellacasa and Baraitser both work with a feminist care ethics concept of care as ‘inseparably a vital affective state, an ethical obligation and a practical labour’ (Puig de la Bellacasa, 2012:197), but they work out from here along different disciplinary lines. Puig de la Bellacasa develops her concept of care through her engagement with science and technology studies and through her research into the practices that are needed to maintain specific and more than human life-worlds. Through her studies of the processes that maintain webs of life, she unearths the temporal dimension of care ‘as the fostering of the endurance of objects over time’ (Puig de la Bellacasa, 2017:171). When considering the question of how to foster the endurance of interdependent worlds that tend to be neglected by productivist temporal regimes, instead of pitting time against care, Puig de la Bellacasa looks at how we might develop relations of care that ‘make time’ differently. Time and temporality viewed in this way, is ‘not just imposed by an epoch or a dominant paradigm but rather made through sociotechnical arrangements and everyday practices’ (Puig de la Bellacasa, 2017:175). Baraitser is invested in thinking about how the gesture ‘to care’, can be one that actually ‘gives time’ (Baraitser, 2017:17). In her book, *Enduring Time*, this is theorised through durational psychosocial practices that fall radically outside of the time of normative development or historical progression. She asks ‘what might it mean to deliberately try to think about staying, inertia, lack of the flow of time, lack of obvious forms of action or psychosocial change, precisely as a way to understand care…?’ (Baraitser, 2017:11).

The historiographer Harry Harootunian writes that the true object of history-writing has always been the nation state and its process of its evolution across the centuries (Harootunian, 2004). Anything that resists being understood through the same categories (of reason and unreason, progression and failure to progress) tends to be eliminated from history altogether or consigned to the domain of contingency. Caring is one of those activities that resists being assimilated into the usual forms of historical representation. It is a kind of work whose time is not often accounted for, not even in the historizing of professions whose whole labour has supposedly been in the service of care of one kind or another. Social work is no exception to this. Its history tends to be told through the drama of its turbulent rising and falling in time with the rising and falling of Western nation states (Rogowski, 2010; Chapman & Withers, 2019; Lavalette, 2011; Jones, 2014). By comparison, the immeasurable durations of ordinary care labour that have been needed just to reproduce ‘social work’ in countless different locations over the years can be dropped from history altogether, or made to appear as though contingent to dramas unfolding at the level of the nation state. Where care is historicized, it often takes the form of a catastrophic failure either to care at all, or to care enough, referring back to the state for a timeline that can produce some kind of rational explanation for how such a thing can have been allowed to happen. As an activity that almost always takes place out of the light of the public, to where the narrating of the passing of time is not so possible, care work very easily falls short of the minimum quotas of eventfulness and ‘rational content’ needed for it to become properly ‘historical’.

In her 2015 paper, *Abyssal Intimacies and Temporalities of Care*, Astrid Schrader reminds us of the distinction, helpful in all types of work involving the making of calculations about care, between caring *for* and caring *about* something or someone. ‘One entails the often-gendered labour of caring for somebody in need, and the other alludes to an affective relation, or caring about’ (Schrader, 2015:667). Schrader explains that caring for somebody is usually goal-oriented as it involves an effort to improve their situation. In these caring relations, ‘the receiver of care is often defined through a lack of ability or autonomy’. To care for them means being concerned about what threatens or might transform the limits that define (them)’ (2015:667). Caring about something on the other hand is a type of care that does not necessarily issue from a specific need or lead to any specific action. It implies more a feeling state in relation to a reality that is not your own but that you are being affected by. It might better be described as a way of living on in the consciousness of something to which you have become sensitised and receptive towards, even when it might be both easier and less painful for you to become detached from it altogether. You could say that both modes: of caring for, and caring about, are utterly necessary to each other and to social work as a whole, but whereas one rests on the idea that social care labour is keeping something going if only for another hour or a day, neither can offer any guarantee that they will necessarily lead to anything being improved or ‘moved on’. Much of social work’s everyday life is concerned with discharging its responsibilities under the first category: that of taking specific action in response to specific need. At another level though, if social work is really to be a caring profession in any meaningful sense, it needs its workforce to take care of realities in relation to which there may be no direct action to take, other than to go on being aware of them and being a part of them – in other words, to not seek to extricate or divest themselves from the time that caring about these realities makes necessary.

Caring, as Puig de la Bellacasa writes, is ‘more than an affective-ethical state: it involves material engagement in labours to sustain interdependent worlds, labours that are often associated with exploitation and domination’ (Puig de la Bellacasa, 2012:198). This is not about calculating how much time might be needed for social workers to stay alive to the potential for things to go wrong in other people’s lives. It is about seeing the work that is already involved in continuing to be implicated for better or for worse in what happens to the ‘interdependent worlds’ that social workers have helped to create. From this perspective, to continue caring about how they are going and about what is becoming of them is really part of the essential labour of social work because not to do so, would be to neglect the relations that made care possible in the first place.

Lisa Baraitser includes ‘maintaining’ along with other practices of care like staying, repeating, delaying and waiting, that are ‘durational’ in that they require time to be in some sense endured. ‘These durational practices are forms of labour that maintain the material conditions of ourselves and others, maintain connections between people, people and things, things and things, people and places, and social and public institutions, along with the anachronistic ideals that often underpin them, and that constitute the systems of sustenance and renewal that support ‘life’ (Baraitser, 2017:49). In thinking of social work as a kind of maintenance work, I am thinking of how so much of what is involved in carrying a social work caseload has to do with sustaining a person, a relationship, a family, a caring arrangement, housing or funding or something else. This must include endless care planning, reviewing and practical things like the filling out of benefit forms and the transportation of children and family members to places where they can be together. But this could also include the tasks that are becoming more arduous and more time consuming as a result of a system that works by endlessly displacing, delaying and interrupting its flow of welfare provision; the keeping up to date of electronic record systems, the email updates, the longer waits over the phone to the Department for Work and Pensions, the deferred managerial decision making, the following up on referrals to other agencies for harder to access specialist care or extra support. All of these ordinary tasks seem to require that the social worker stays stuck in repetitive cycles of checking, updating, waiting and going back and forth and yet, as Baraitser observes, the labour of maintenance is also a source of renewal because in a very real and material way, this is the sort of work that is necessary for everyday life to keep being renewed (2017:50).

## Enduring painful time in social work now

As we have seen, there is no way of extracting social work from its original attachments that is not going to be in some sense painful to endure. It seems that however we want to manage the past of social work, we end up having to retrench on decades-long attachments to what has been ‘good’ about it. Even if I cannot fully agree with them, I can relate to Rogowski’s despair over the ‘fall’ of social work. I understand why Brady, Sawyer and Perkins might choose to embrace the belief that social work is better off without radical aspirations. I can share Chapman and Withers’ concern over the imperial timeline of social work with its legacy of violent care. My aim has not been to criticise these recent histories of social work, but to complicate them by reading them as stories that assemble the historical present and enact their own modes of getting through it. Without contributing anything in particular to our sense of where social work as a historical project is going now, or how exactly it has failed to live up to its own expectations, I have tried to suggest another way of relating to time in social work by turning from the work of assembling a painful present, towards the task of enduring it in everyday life. This involves relocating its work to the phenomenal present. Harootunian writes about this mode of thinking time that it is not simply ‘the site of punctual events’ but ‘a durational moment and its subsequent ontologization’ (Harootunian, 2004:184). The effect of its emphasis on the present is to ‘disclose the recognition of the existence of different temporalities between history (especially ‘national history’) and the everyday’ (2004:183). This is the recognition that the present as lived out through everyday life can potentially break with all antecedents, to offer a new way to envision the relationship between the present and the past. My suggestion is that by anchoring social work in durational time, we might be better positioned to think from within the painful present about what it means to be temporally involved in social care work now.

Social work seen through the optic of the everyday is arguably already well practiced at living with the problems of trying to do the work of maintaining (bodies, relationships, worlds) within suspended painful time. Hage writes about the experience of living in this state, that it presumes a lack of agency on the part of the person for whom making progress has become an impossible task. ‘Indeed, it is a lack of agency that defines stuckedness whether physically or existentially understood’ (Hage, 2009:100). This can be typical of how time often feels in social work. Afterall, social work care labour usually only begins after all the other options for working through a difficult time have been exhausted or broken down, and its purpose is often to assist in finding ways to work through matters of the kind that will probably never completely resolve. When for example, somebody finds that they are unable to live in their own home safely following a decline in their physical or mental health, or when a carer finds themselves unable to go on offering an essential care work, often it is not even clear to those involved what sort of change is needed or desired, or by whom. There is also something ‘intrinsically ambiguous’ about the practice of social work on a relational level (Roose, Roets & Bouverne-De Bie, 2012:2). Not only can it never obtain a clear-cut solution for social problems which are complex and multidimensional by nature, but social care work very often intersects with intergenerational timelines of social injustice. Its arduousness is compounded by the need to practice what Baraitser describes as ‘the maintenance of relations with ourselves and others through histories of oppression that return in the present again and again’ (Baraitser, 2017:54). In all these different ways, the labour of social work shows its commitment to staying in contact with realities which are in some sense stuck, suspended or recurring. This way of living the present may well be difficult at times because it assumes that only by continuing to endure the partial collapse of relations, plans, arrangements, worlds, histories, can social workers stay in a caring relation to them. It is not hard to see why such a relation might end up becoming more than what can be endured.

To the extent that care involves a commitment to ways of going on without any goal in mind, its labour often implies what Baraitser has termed a way of being ‘without project’. She writes that, ‘to be ‘without project’ is to live in a form of time that does not define itself in relation to a projection into an open libidinal future – a way of being in time that is not about going anywhere, and is not about going nowhere, but is perpetually concerned with what is produced…’ (Baraitser, 2017:61). That ‘there is no way to reveal this time other than to live it’ is a circumstance shared, though in a superficial sort of way with the stuck presents of Rogowski, Chapman & Withers and Brady, Sawyer & Perkins. In them, social work appears to have lost its orientation to a project unfolding in historical time and entered a phase of depression, withdrawal and disavowal in relation to the things that it once cared about. In the phenomenal present of social work however, to be sometimes without a project of one’s own may be the condition of what makes any sort of care possible. This implies a suspension of social work’s projections for its own time and the endurance of what may be painful about trying to continue to honour caring engagements, that probably will not lead to anything or to anywhere in particular.

## Footnotes

1 Mark Fisher writes about the temporality of late capitalism that it subsumes and consumes all of previous history into a ‘system of equivalence’ (2009:8). His notion of capitalist realism entails subordinating oneself to this temporality as though it were the one true reality.
